# A False-Positive Case of Methylmalonic Aciduria by Tandem Mass Spectrometry Newborn Screening Dependent on Maternal Malnutrition in Pregnancy

**DOI:** 10.3390/ijerph17103601

**Published:** 2020-05-20

**Authors:** Claudia Rossi, Ilaria Cicalini, Cristiano Rizzo, Mirco Zucchelli, Ada Consalvo, Silvia Valentinuzzi, Daniela Semeraro, Giorgia Gasparroni, Patrizia Brindisino, Diego Gazzolo, Carlo Dionisi-Vici, Vincenzo De Laurenzi, Damiana Pieragostino

**Affiliations:** 1 Center for Advanced Studies and Technology (CAST), University “G. d’Annunzio” of Chieti-Pescara, 66100 Chieti, Italy; ilaria.cicalini@unich.it (I.C.); m.zucchelli@unich.it (M.Z.); ada.consalvo@libero.it (A.C.); silvia.valentinuzzi@unich.it (S.V.); d.semeraro@unich.it (D.S.); delaurenzi@unich.it (V.D.L.); or dpieragostino@unich.it (D.P.); 2 Department of Medicine and Aging Science, University “G. d’Annunzio” of Chieti-Pescara, 66100 Chieti, Italy; diego.gazzolo@unich.it; 3 Metabolic Diseases Unit, Bambino Gesù Children Hospital and Research Institute, 00165 Rome, Italy; cristiano.rizzo@opbg.net (C.R.); carlo.dionisivici@opbg.net (C.D.-V.); 4 Department of Medical, Oral and Biotechnological Sciences, University “G. d’Annunzio” of Chieti-Pescara, 66100 Chieti, Italy; 5 Department of Pharmacy, University “G. d’Annunzio” of Chieti-Pescara, 66100 Chieti, Italy; 6 Neonatal Intensive Care Unit, University “G. d’Annunzio” of Chieti-Pescara, 66100 Chieti, Italy; giorgia.gasparroni@gmail.com (G.G.); patrizia.brindisino@asl2abruzzo.it (P.B.)

**Keywords:** organic acidemias, newborn screening, methylmalonic acidemia, false-positive, mass spectrometry, vitamin B12 deficiency, LC-MS/MS, maternal defects

## Abstract

Methylmalonic Acidurias (MMAs) are a group of inborn errors of metabolism (IEMs), specifically of propionate catabolism characterized by gastrointestinal and neurometabolic manifestations resulting from a deficiency in the function of methylmalonyl-CoA mutase, methylmalonyl-CoA epimerase, and cobalamin metabolism. In Expanded Newborn Screening (NBS), increased levels of propionylcarnitine (C3) and/or of its ratios by MS/MS analysis of dried blood spots (DBS) samples are suggestive for either Propionic Acidemia or MMAs. C3 elevation is not considered a specific marker for these disorders, resulting in high false-positive rates. The use of analyte ratios improves specificity, but it still cannot resolve the diagnostic issue. Second-tier testing are strongly recommended as confirmation of primary NBS results and for a differential diagnosis. LC-MS/MS analysis allows the quantification of more specific markers of the disorder. Here, we report the case of a newborn with a suspected MMA at Expanded NBS and at second-tier test. Given the urgent situation, in-depth diagnostic investigations were performed. Further investigations surprisingly revealed a Vitamin B12 deficiency due to a maternal malnutrition during pregnancy. This case emphasized that metabolic alterations at NBS may not only be influenced by genome and related to IEMs, but also to external factors and to maternal conditions.

## 1. Introduction

Organic acidemias (OAs), also known as organic acidurias, are a class of inherited disorders of intermediary metabolism resulting primarily from deficiencies of specific enzymes or transport protein in one of the several metabolic pathways of the catabolism of amino acids (AAs), carbohydrates, or lipids. Since organic acids occur as physiologic intermediates in these pathways, the enzyme/protein deficiency results in abnormal, and usually toxic, accumulation of organic acids in the body and their excess is excreted in urine [1,2]. Among OAs, Propionic Aciduria (PA), Methylmalonic Aciduria (MMA), Isovaleric Aciduria (IVA), and also Maple Syrup Urine Disease (MSUD) are classified as the most common inborn errors of branched chain AA (BCAA) catabolism and are referred to as “classical OAs”. OAs generally present high anion gap metabolic acidosis with hyperammonemia [1,3,4]. Interestingly, a smart acronym, VOMIT, is often used to summarize both the OA characteristic biochemical finding (valine, odd chain fatty acids, methionine, isoleucine, and threonine) and the initial clinical one of vomiting [1].

**Figure 1:**
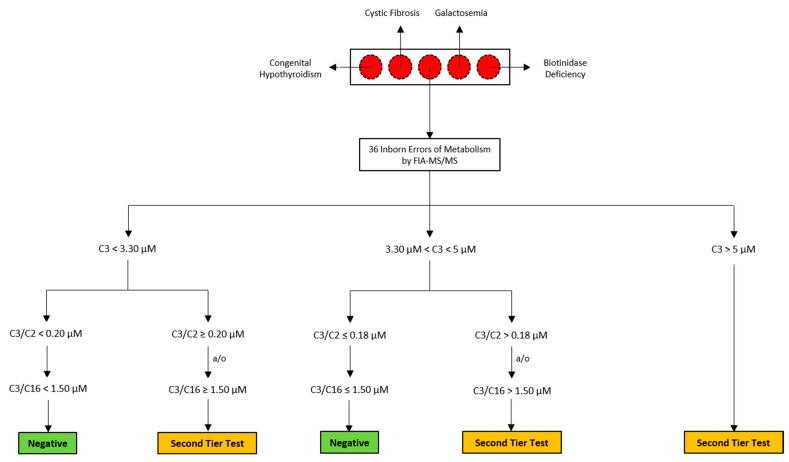
Workflow of the second-tier test strategy in the management of C3 alterations.

MMA disorders are a heterogeneous group of inborn errors of metabolism (IEMs), specifically error of propionate catabolism characterized by gastrointestinal and neurometabolic manifestations. Accumulation of methylmalonic acid (mma) in body fluids and tissues is a biochemical characteristic of MMA. MMAs can be classified as isolated MMAs and can be related to an isolated deficiency in propionyl-CoA catabolism, or as combined MMAs, in combination with other inherited metabolic defects [1,2,4]. In particular, the isolated MMA may be due to a complete or partial defect of the enzyme methylmalonyl-CoA mutase MCM, a deficiency in the transport or synthesis of its cofactor (adenosyl-cobalamin), or by a defect of the enzyme methylmalonyl-CoA epimerase [5]. Clinical signs and symptoms of MMA usually appear within the first few days or weeks of life and may also be accompanied by intermittent life-threatening acute metabolic decompensations [6], even if a later-onset form may present later during the first years of life [4]. Newborn Screening (NBS) allowed for early diagnosis and for a better estimation of the incidence of MMA. The detection rate of isolated MMA was < 1 per 100,000 newborns in all regions, with the exception of the Middle East and North Africa where it is higher, close to 6 per 100,000 newborns. Instead, for Asia-Pacific, Europe, and North America, the incidence of MMA (all types) per 100,000 newborns is 0.79, 1.12, and 1.22, respectively [5]. In MS/MS NBS, screen-positive cases for MMAs or PA are identified by revealing increased levels of propionylcarnitine (C3) and/or of its ratios [7]. It is well known that C3 elevation is not considered a specific marker for these disorders, resulting in high false-positive rates. Furthermore, the use of analyte ratios such as C3/acetylcarnitine (C3/C2), C3/palmitoylcarnitine (C3/C16) improved specificity, but it still cannot resolve the diagnostic issue and differentiate between PA and MMAs, still giving a high number of false positive. Thus, second-tier testing of all screen-positive cases are strongly recommended to decrease the recall rate due to false positive results in the primary screening test to eventually confirm primary NBS results and for a first differential diagnosis. Second-tier tests are usually performed by Liquid Chromatography coupled to MS/MS (LC-MS/MS) and are able to identify additional analytes, not measured in primary NBS test, as more specific markers of the suspected disease, leading to a better interpretation of the abnormal results [8]. In case of suspected MMAs or PA, LC-MS/MS analysis allows the quantification in the same NBS specimen of mma, methylcitric acid (mca), and eventually homocysteine (hcy) as more specific markers of the diagnostic suspicion, without the necessity to additionally contact the newborn’s family. The introduction of second-tier testing for the suspicion of MMAs or PA is considered an important strategy to cope with C3 false positive results at NBS [8]. Figure 1 shows the workflow of the second-tier testing strategy in the management of C3 alterations. In recent years, the application of MS/MS to NBS for the expansion of the IEMs panel has strongly changed the scenario: leading to significant advantages in terms of diagnosis and treatment of these metabolic disorders, but also bringing out a number of “side effects” [9]. In detecting newborns with classical OAs in a pre-symptomatic or early symptomatic period, MS/MS NBS may point out other conditions as unintended consequences: partial enzyme deficiency, maternal defects, nutritional deficiency, drugs, prematurity, and a sick neonate [4].

Here, we report the case of a newborn with a suspected MMA following the alterations at expanded NBS and the increased levels of mma and hcy at the second-tier test, which doubled in a couple of days. Given the urgent situation and considering the increasing marker alteration, in-depth diagnostic investigations by further specific biochemical tests were performed. Despite the strong suspicion of a form of MMA, further investigations surprisingly revealed a vitamin B12 (Vit. B12) deficiency due to a maternal malnutrition during pregnancy.

This case emphasized that metabolic alterations at expanded NBS may be influenced not only by genome and be related to IEMs, but also to external factors and secondary to maternal conditions.

## 2. Case Presentation

### 2.1. Clinical Presentation

We report a case of a 9-day-old male Moroccan newborn admitted to our third level referral center for neonatal intensive care (NICU) from a spoke center on 4 May 2019. The male baby was born at 41 weeks of gestation from natural childbirth, with a weight of 2,920 kg. Further details of standard and clinical monitoring parameters are reported in Table S1 of Supplementary Materials. We suspected a metabolic disease due to altered expanded newborn screening characterized by increased levels of C3, C3/C2, C16:1OH\C17 (3-Hydroxy-hexadecenoylcarnitine), and methionine Met.

At admission to NICU, there was no overt organ perinatal disease, fetal-neonatal growth parameters were within normal ranges according to local standards, and neurological evaluation showed axial hypotonia previously diagnosed at birth. No other isolated symptoms were detectable.

Standard monitoring parameters such as heart and respiratory rates and blood pressure arterial oxygen saturation were within normal ranges at the age under investigation.

Standard laboratory parameters, listed in Supplementary Materials Table S2, as well as electrolytes and partial pressure of carbon dioxide (pCO2) levels were within normal ranges at different monitoring time-points whilst metabolic pattern was suggestive of low chobalamine (83 pg/mL) and high whole blood mma and hcy levels, respectively, as described below. Conversely, amino acids (AAs), C2, and urinary fatty acids were within normality ranges at the stage under investigation.

Furthermore, from careful family history recording, it emerged that the maternal diet throughout pregnancy avoided meat, milk, and milk derivatives, thus causing an important lack of adequate intake of macro and micronutrients for the fetus. The fact was corroborated by low vitamin B12 plasma levels (44 pg/mL) at six days from the infant’s recovery.

On the basis of the aforementioned data, we provided supplementation to the infant through cianocobalamine (1000 mcg i.m. single dose), achieving fast and complete resolution of neurological symptoms and discharge of the newborn at five days from NICU admission.

### 2.2. Routine Newborn Screening Analysis and Second-Tier Testing

In agreement with laboratory collection protocol, samples for NBS are collected at 48–72 h after birth by heel-pricking blood specimen onto Ahlstrom 226 filter paper provided by PerkinElmer. Details of NBS by a flow injection-tandem mass spectrometry analysis (FIA-MS/MS) for the detection of 36 IEMs [9] as well as information on second-tier analysis for the determination of mma, mca, and hcy by LC-MS/MS are fully described in Supplementary Materials and specifically reported in Figure S1 and in Tables S3–S6.

### 2.3. NBS Analysis and Second-Tier Testing of The Suspected Neonate

**Figure 2:**
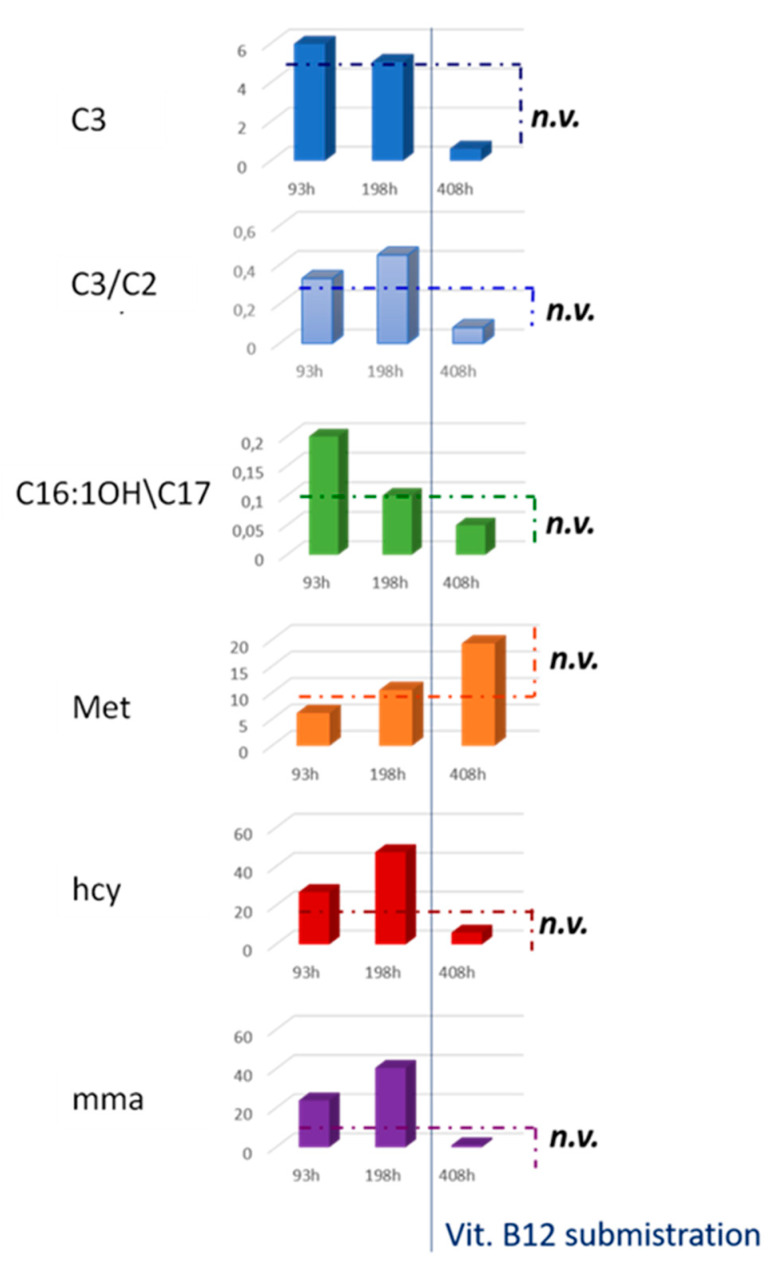
Histograms in the figure show the levels of C3, C3/C2, C16:OH\C17, Met, hcy, and mma from MS/MS NBS and the second-tier test of the newborn at 93, 198, and 408 h of life.

The first DBS sample (64 h of life) was not sufficient to undergo reliable NBS analysis and the screening test revealed elevated C3, C3/C2, C3/Met, C16:1OH\C17, and 3-Hydroxy-octadecadienoylcarnitine (C18:2OH) levels. Because of the insufficient specimen quality, the birth center was recalled to repeat the sampling (4 days of life) and 24 h later, the NBS analysis confirmed the increased levels of C3, C3/C2, C3/Met, C16:1OH\C17, and C18:2OH, also showing decreased Met concentrations. Figure 2 and Table 1 summarize the data from MS/MS NBS and second-tier tests of the neonatal clinical case we described. Since the newborn presented C3 > 5.00 μmol/L (5.99 μmol/L) at the first screening analysis, a second-tier test was performed to determine hcy, mca, and mma concentrations to further investigate the diagnostic suspicion. The second-tier test turned out to be positive, with hcy = 26.9 μmol/L (normal value, nv < 10 μmol/L), mma = 23.9 μmol/L (nv < 4 μmol/L), and mca within reference limits. The newborn was entrusted to clinicians from Bambino Gesù Children’s Hospital for further confirmatory analysis, while the NBS laboratory asked for new DBS samples. In the meantime, the newborn was transferred to another hospital equipped with a NICU in Abruzzo. The new DBS sample (9 days of life) was extracted for NBS analysis, confirming the alteration for C3, C3/C2, and C16:1OH\C17. Following this, the C3 s-tier test revealed an even stronger increase in the metabolites levels: hcy = 47.4 μmol/L (nv < 10 μmol/L), mma = 40.5 μmol/L (nv < 4 μmol/L) and mca = 1.2 μmol/L (nv < 1 μmol/L).

### 2.4. Biochemical Diagnostic Confirmations

Positivity in newborn screening must be followed by confirmatory analyses. This includes the analysis of urinary organic acids, plasma acylcarnitines (ACs), and plasma AAs according to the suspected metabolic disorder. Recommended confirmation testing procedure for positive newborn screens due to increased C3 is organic acid analysis [10].

Urinary organic acid are extracted with ethyl acetate, derivatized with N,O-Bis(trimethylsilyl)trifluoroacetamide (BSTFA), and analyzed by capillary gas Chromatography-mass spectrometry (GC-MS) analysis [11].

**Figure 3:**
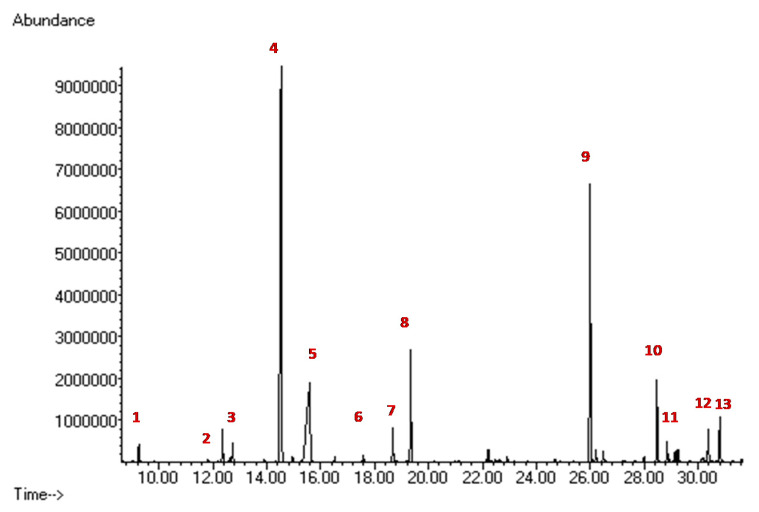
GC-MS Urinary organic acid profile. (**1**) Lactic acid; (**2**) 3-hydroxypropionic acid; (**3**) 3-Hydroxy butyric; (**4**) mma; (**5**) urea; (**6**) succinic acid; (**7**) fumaric acid; (**8**) 2-phenylbutiric acid (internal standard1); (**9**) 2-ketogluraric acid, (**10**) tricarballylic acid (internal standard 2); (**11**) cis-aconitic acid; (**12**) citric acid; (**13**) hippuric acid.

Our patient showed an altered GC-MS profile of urinary organic acids with a marked increase in mma 868 mmol/mol creatinine (nv < 7 mmol/mol creatinine) (Figure 3).

Despite the biochemical findings of the diagnostic confirmations, the clinical evaluation of the newborn and further insights on maternal dietary management surprisingly revealed a vitamin B12 deficiency due to a maternal malnutrition during pregnancy, allowing an early treatment with vitamin B12. After 14 days from the beginning of the treatment, a new DBS sample was collected for a further NBS test and eventual second-tier analysis.

Finally, the newborn metabolic profile by MS/MS NBS was normal. Anyway, the C3 s-tier testing was performed and interestingly revealed no alterations in the levels of hcy, mma, and mca. In particular, after Vitamin B12 treatment, the following values were measured for NBS markers by FIA-MS/MS: C3 = 0.62 μmol/L (nv < 5.0 μmol/L), C3/C2 = 0.08 (nv < 0.20), and C16:1OH\C17 = 0.05 μmol/L (nv < 0.09 μmol/L). The second-tier test showed normal values for each marker: hcy = 6.1 μmol/L (nv < 10 μmol/L), and mma and mca were both below their limit of quantification.

## 3. Discussion

The expansion of neonatal screening programs has not only increased the number of eventually diagnosed congenital metabolic errors, but has also increased the number of false positive results [12]. One of the analytes that has caused numerous false positive tests is C3, the junction point of numerous catabolic pathways of BCAAs, Met, threonine, and odd chain fatty acids.

In order to improve the interpretation of the NBS results test, the ratio between C3 and C2 and between C3 and palmitoylcarnitine (C16) are often also evaluated, in addition to the absolute levels of C3. C16:OH\C17 levels also provide useful information about the suspected diagnosis of PA or MMAs, recently recognized as novel candidate biomarkers for PA and MMAs [13]. All metabolites and ratios monitored during the first level screening test, if altered, still require a second level test to confirm the diagnostic suspicion [12].

**Figure 4:**
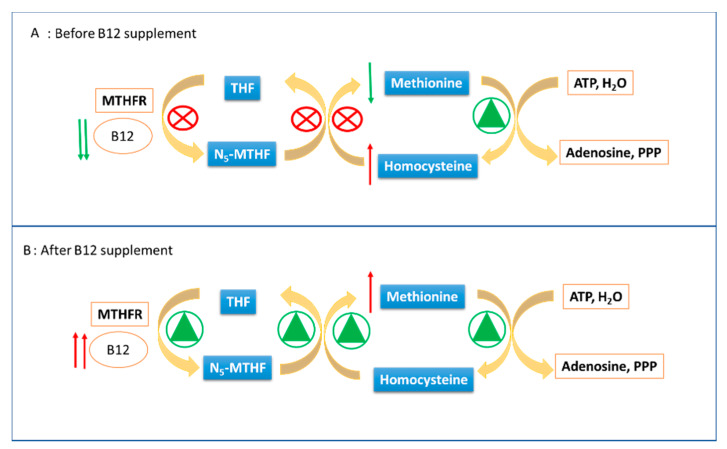
Met and hcy metabolic pathway before and after Vit. B12 supplementation. Methylenetetrahydrofolate reductase = MTHFR; THF = tetrahydrofolate; Adenosine 5′-Triphosphate = ATP; N_5_- methyltetrahydrofolate = N_5_-MTHF; Triphosphate = PPP.

Here, we present a case report of a positive result in newborn screening to first analysis, to second tier test and to urinary confirmation test, but which results in a negative result during the follow-up after Vit. B12 supplement. Data shows the simultaneous increase of mma and hcy, two molecules linked only by the fact that they are metabolized by Vit. B12 dependent enzymes, supporting the hypothesis of the lack of such a cofactor. Actually, as reported, both the mother and the newborn show low levels of Vit. B12. Moreover, Vit. B12 supplement allowed the drastic reduction of mma and hcy at the second tier test and, at the same time, the return to normal of screening on DBS, with levels of C3, C3/C2, C3/C16, and C16:OH\C17 below the reference limits, which is also shown in Figure 2. Taken together, these data allow one to exclude the diagnostic hypotheses of MMA, MCA, and even Vit. B12 absorption defects, since Vit. B12 becomes available to enzymes, as demonstrated by the lowering of the metabolites under study. Met levels contribute to reinforce this interpretation. This metabolite is low during Vit. B12 deficiency since it is efficiently transformed into hcy (which increases accordingly), but which is not effectively reformed due to inefficiency of the methylenetetrahydrofolate reductase (MTHFR) enzyme (which needs Vit. B12 as a coenzyme). Immediately after the Vit. B12 supplement, the MTHFR enzyme resumes its activity, transforming the large amount of hcy back into Met, which soon increases beyond normal levels after the supplementation, as reported in Figure 4.

The described case certifies as Vitamin B12 deficiency due to the fact that a maternal malnutrition during pregnancy is reflected in the metabolic pathway of newborns, representing an interesting and confounding factor. In particular, in the present case, maternal malnutrition was related to socio-economic issues rather than dietary preferences. The hypothesis of malnutrition and the consequent vitamin deficiencies could be strengthened by the dosage of Vitamin D and calcium homeostasis, two factors that are also affected by the lack of dairy products, which is typical of vegans or vegan like feeding [14].

It is worth noting that the spread of MS/MS NBS, through the detection of maternal nutritional vitamin B12 deficiency, highlighted the high frequency of cobalamin deficiency [15]. Interestingly, the NBS metabolic profile may identify neonatal and maternal vitamin B12 deficiency by revealing the alteration of C3 and of mma and hcy at the second-tier test [16]. These data suggest that vitamin B12 deficiency, a common aspect of many conditions, is more frequent than previously thought, also pointing out that non-inherited conditions may be secondary to maternal defect [17,18].

Therefore, it is strongly recommended that special dietary maternal regimens on neonatal cards are indicated to suggest plasma Vitamin B12 quantification for newborns and mothers in the case of mild alteration at the second-tier test. Moreover, following Vitamin B12 supplementation, a careful follow-up of the newborn is required to avoid possible Vitamin B12 absorption defect.

Anyway, it is worth noting that this maternal condition is not only detectable by NBS. As is well known, metabolic findings and alterations at NBS may be influenced by genome as well as by maternal defects and external factors, including drugs and diet [15,19]. In fact, the detection of maternal deficiencies is now well recognized as a complicating factor in MS/MS NBS, primarily because most maternal defects may present without major symptoms [Ref]. Interestingly, NBS findings showed that newborns with decreased levels of free carnitine were in some cases associated with maternal primary carnitine defect [8,20,21,22] and in some other cases, with maternal glutaricacidemia type I [20,23]; newborns with increased levels of 3-hydroxyisovalerylcarnitineassociated were sometimes associated with asymptomatic mothers with 3-methylcrotonyl-CoA carboxylase deficiency [8,15,20,24,25]; and newborns with elevated values for phenylalanine with mothers affected by undiagnosed mild hyperphenylalaninemia [8]. All the aforementioned cases highlight the importance of follow-up testing of the mother [15].

## 4. Conclusions

The present case is a testimony of how maternal B12 deficiencies could be reflected in metabolic alterations, even serious ones, in newborns, with profiles perfectly superimposable to IEMs, and profiles even confirmed in second-tier and in urinary confirmation tests. Moreover, the accumulation of toxic metabolites increases if newborns are breastfed, leading to a condition that may dramatically reflect pathological signs and symptoms. Therefore, in our opinion, monitoring mother and offspring B12 levels, in the case of vegan and vegetarian diets, could be strongly recommended in order to avoid metabolic abnormalities such as those considered in this case report. It should also be considered that infants born from mothers that follow plant-based diets are at risk of nutritional deficiencies such as protein, iron, vitamin D, calcium, iodine, omega-3, as well as vitamin B12. These data could even suggest that one carry out a complete vitamin panel in those infants born to mothers who follow a vegan-like diet, in order to avoid both enzymatic malfunctions and false positives to NBS, since newborns show a metabolic profile close to toxicity as early as 48 h after birth.

## Figures and Tables

**Table 1:** Results of NBS and second level tests carried out at 93, 198, and 408 h from birth. Results of the test carried out after Vit. B12 treatment are highlighted in bold in the table.

Analyte	Age at Birth (Hours)			Normal Value
	93 h	198 h	408 h	
**C3**	5.99	5.06	**0.62**	<3.3
**C3/C2**	0.33	0.45	**0.08**	<0.18
**C16:1OH\C17**	0.2	0.1	**0.05**	<0.09
**Met**	6.16	10.5	**19.3**	7-37
**hcy**	26.9	47.4	**6.1**	<10
**mma**	23.9	40.5	**1**	<4
**mca**	<1	1.2	**<1**	<1

## Supplementary Materials

The following are available online at https://www.mdpi.com/1660-4601/17/10/3601/s1, Figure S1: Chromatographic separation of hcy, mma and mca by LC-MS/MS analysis, Table S1: Standard clinical and monitoring parameters of the suspected newborn, Table S2: Standard laboratory parameters of the suspected newborn, Table S3: Concentration levels (µM) for calibrators and QC materials for LC-MS/MS analysis of hcy, mma and mca. SS1= 10 mM, and SS2= 1 mM, Table S4: Multiple Reaction Monitoring (MRM) functions and settings for detection of hcy, mma and mca are shown, Table S5: The Linearity criteria of the method were evaluated by analyzing the DBS samples fortified with increasing concentrations of hcy, mma and mca for five consecutive days. For each analytical session, the linear regression coefficient R2 was assessed. The method proved linear with R^2^> 0.994 for hcy, R^2^> 0.995 for mma, R^2^> 0.998 for mca. Average values, both for calibration parameters with the respective standard deviations (SD) and for R^2^, are reported, Table S6: The method reproducibility, accuracy and precision were assessed, examining the Mean Value, Standard Deviation and CV% of low and high QC.
