# Supplementary material for: A False-Positive Case of Methylmalonic Aciduria by Tandem Mass Spectrometry Newborn Screening Dependent on Maternal Malnutrition in Pregnancy
Source: Int J Environ Res Public Health. 2020 May 20;17(10):3601. doi: 10.3390/ijerph17103601 (PMC7277087; doi:10.3390/ijerph17103601)
Supplement: Supplementary file 1 [file ijerph-17-03601-s001.pdf]

Supplementary Material of:

A false-positive Case of MethylmalonicAciduria by Tandem Mass SpectrometryNewborn Screening Dependent On Maternal Malnutrition in Pregnancy

Claudia Rossi [1,2*], Ilaria Cicalini [1,2], Cristiano Rizzo [3], Mirco Zucchelli [1,4], Ada Consalvo [1,4], Silvia Valentinuzzi [1,5], Daniela Semeraro [1,2], Giorgia Gasparroni [6], Patrizia Brindisino [6], Diego Gazzolo [2,6], Carlo Dionisi-Vici [3], Vincenzo De Laurenzi [1,4] and Damiana Pieragostino [1,4]

1 Center for Advanced Studies and Technology (CAST), University "G. d'Annunzio" of Chieti-Pescara, 66100 Chieti, Italy; ilaria.cicalini@unich.it (I.C.); m.zucchelli@unich.it (M.Z.); ada.consalvo@libero.it (A.C.); silvia.valentinuzzi@unich.it (S.V.); d.semeraro@unich.it (D.S.); delaurenzi@unich.it (V.D.L.); dpieragostino@unich.it (D.P.)

2 Department of Medicine and Aging Science, University "G. d'Annunzio" of Chieti-Pescara, 66100 Chieti, Italy; diego.gazzolo@unich.it

3 Metabolic Diseases Unit, Bambino Gesù Children Hospital and Research Institute, 00165 Rome, Italy; cristiano.rizzo@opbg.net (C.R.); carlo.dionisivici@opbg.net (C.D.-V.)

4 Department of Medical, Oral and Biotechnological Sciences, University ''G. d'Annunzio'' of Chieti-Pescara, 66100 Chieti, Italy

5 Department of Pharmacy, University "G. d'Annunzio" of Chieti-Pescara, 66100 Chieti, Italy

6 Neonatal Intensive Care Unit, University "G. d'Annunzio" of Chieti-Pescara, 66100 Chieti, Italy; giorgia.gasparroni@gmail.com (G.G.); patrizia.brindisino@asl2abruzzo.it (P.B.)

* Correspondence: claudia.rossi@unich.it; Tel.: +39-0871-541596; Fax: +39-0871-541598

Received: 27 April 2020; Accepted: 17 May 2020; Published: date

Keywords: organic acidemias; newborn screening; methylmalonicacidemia; false-positive; mass spectrometry; vitamin B12 deficiency; LC-MS/MS; maternal defects

---

Materials and dried blood spots samples preparation for homocysteine, methylmalonic acid and methylcitric acid quantification by LC-MS/MS analysis

Homocysteine (hcy) and methylmalonic acid (mma) were purchased from Sigma-Aldrich, methylcitric acid (mca) from CDN Isotopes; relative internal standards $^{2}H_{4}$-hcy and $^{2}H_{3}$-mma were provided from Cambridge Isotope Laboratories, Inc and $^{2}H_{3}$-mca from CDN Isotopes.

Stock solution IS1 was prepared by dissolving internal standards in water/acetonitrile 80:20 with 0.4% formic acid to obtain a final concentration of 500 μM for the three ISs. Stock solution IS2 was prepared by diluting 1:50 the IS1 solution with water to obtain a final concentration of 10 μM for the three ISs. Internal standards stock solutions IS1 and IS2 were stored at +4°C. Working solution IS3 was prepared freshly by adding 1 M Dithiothreitol (DTT, purchased from Sigma-Aldrich) to IS2 solution diluted 1:10 with acetonitrile/water 70:30 with 0.5% formic acid. Calibrators and Quality Controls (QCs) were made by spotting onto filter paper some drops of whole blood from healthy donors fortified with different proportions of two standard solutions at a concentration of 10 mM and 1 mM, respectively. Calibrators and QCs were prepared following the indications in Supplementary Tables S3.

For hcy, mma and mca quantification by LC-MS/MS analysis, 250 μL of working solution IS3 were added to two 3.2 mm-DBS disks of sample, calibrators and QCs. Each sample was gently mixed (20°C, 60 min) in a Thermomixer (Eppendorf®). The supernatant was transferred into a new 1.5 mL

tube and dried in a SpeedVac. The residue was then reconstituted with 100 µL of 3N HCl in n-Butanol (purchased from Sigma-Aldrich) and mixed in a Thermomixer (65°C, 15 min). The sample was dried once again in a SpeedVac, then the residue was reconstituted with 100 µL of water, briefly centrifuged and the supernatant was transferred into polypropylene vial (provided by Waters Corporation). The vials were finally placed in the system autosampler for the LC-MS/MS analysis.

The LC-MS/MS system consisted of an ACQUITY UPLC I-Class/Xevo TQD IVD tandem quadrupole mass spectrometer (Waters Corporation, Milford, MA, USA). The system operated in positive electrospray ionization mode using TargetLynx XS software (Waters Corporation, Milford, MA, USA). 5 µL were injected into the ion source and the run time was 9 minutes, injection-to-injection. For UPLC analysis, the ACQUITY UPLC BEH C18 2.1 x 50mm column with ACQUITY UPLC BEG C18 VanGuard pre-column was used. The mobile phase comprised a binary solvent system: H2O (Solvent A) and ACN (Solvent B), both containing 0.1% formic acid. The initial solvent composition, 95% A and 5% B, was maintained for 1.0 minute. The flow gradient profile involved the following steps: increasing from the initial conditions to 90% B within 5.0 min, holding for 1.0 min before coming back to 95% A. The flow rate was 0,5 mL/min and the column was maintained to 40°C. An example of chromatogram is shown in Supplementary Figure S1.

Functions 1-3 in Supplementary Table S4 summarize all the parameters refer to the Multiple Reaction Monitoring (MRM) experiments created for each analyte.

All the information regarding the method reproducibility, accuracy and precision are detailed in Supplementary material Tables S5-S6.

Routinely Newborn Screening Analysis and Second-tier Testing

Dried blood spot (DBS) samples for NBS are punched out into 3.2 mm-disks to perform a flow injection-tandem mass spectrometry analysis (FIA-MS/MS) for the detection of 36 IEMs, including AAs, urea cycle, organic acid and fatty acid oxidation disorders. Actually, four 3.2 mm DBS disks are used to test by immunofluorimetric assays congenital hypothyroidism (CH), cystic fibrosis (CF), galactosemia and biotinidase deficiency, respectively, and the fifth DBS disk is employed for FIA-MS/MS analysis. For the latter, the DBS disk (of approximately 3-3.2 µL whole blood) is extracted for the determination of 14 AAs, 35 acylcarnitines (ACs), free carnitine and succinylacetone, by using the NeoBase 2 Non-Derivatized MSMS Kit (Perkin Elmer Life and Analytical Sciences, Turku, Finland. The FIA-MS/MS system consists of an Acquity UPLC I-Class coupled to a Xevo TQD tandem quadrupole mass spectrometer (Waters Corporation, Milford, MA, USA). The system operates in positive electrospray ionization mode by multiple reaction monitoring (MRM) acquisition. 10 µL are injected into the ion source and the run time is 1.1 min, injection-to-injection. Data are finally processed by MassLynx V4.2 and NeoLynx Software (Waters Corp.).

Second-tier test for the quantification of methylmalonic acid, methylcitric acid and homocysteine by LC-MS/MS

For C3 second tier test, the DBS sample is punched out twice into final diameter disks of approximately 3.2 mm, using an automatic puncher. Two DBS disks (equivalent to approximately 6-6.4 µL whole blood) are extracted for the determination of mma, mca and hcy by LC-MS/MS (Figure S1). Details of C3 second-tier analysis by LC-MS/MS are fully reported in in Table S3-S6.

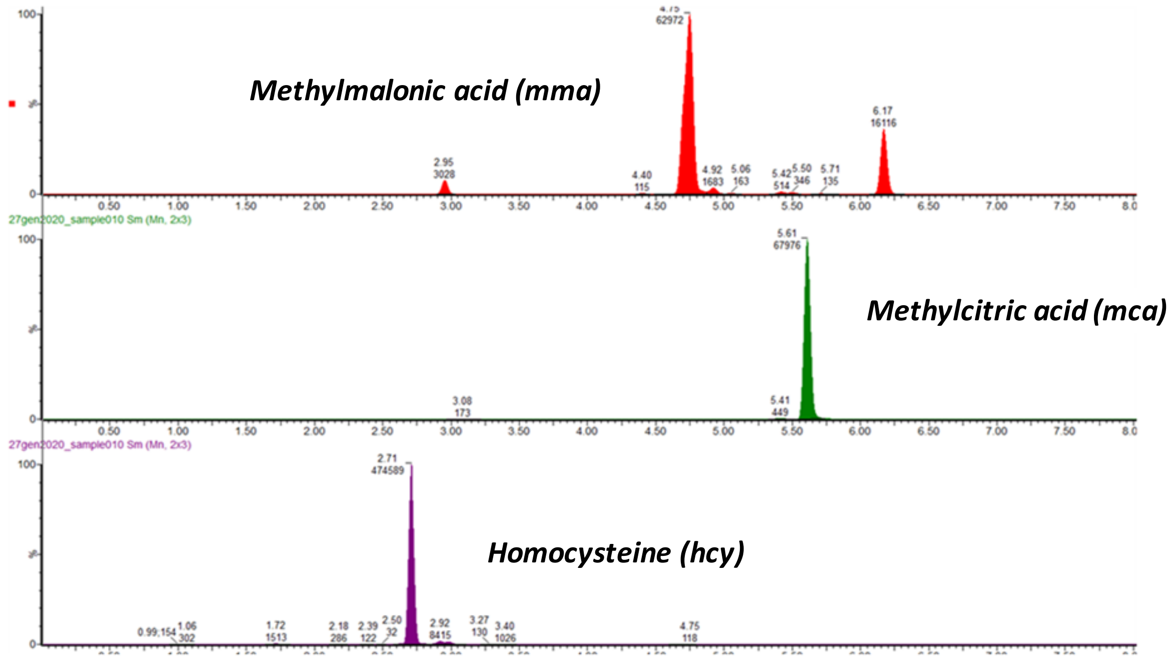

Figure S1. Chromatographic separation of hcy, mma and mca by LC-MS/MS analysis.

Table S1. Standard clinical and monitoring parameters of the suspected newborn.

**Table S1.**	
Birth weight	2,920 Kg
Birth length	50 cm
Head circumference	33 cm
Gestational age	41 w+3 d
Heart rate	140 bpm
Respiratory rate	40 RR
Peripheral oxygen saturation	100%

Table S2. Standard laboratory parameters of the suspected newborn.

**Table 2**	
	**Value (Normal range)**
Red cells	5.76 x 10^3/mmc (3.60 – 6.20)
Hemoglobin	18.2 g/dL (12.5 -20.5)
Hematocrit	55.3% (39.0 – 63.0)
White blood cells	11.28 x 10^3uL (4.50 – 13.00)
Neutrophils	3.06 x 10^3uL (1.70 – 4.10)
Lymphocytes	5.78 x 10^3uL (3.10 – 7.80)
Monocytes	1.78 x 10^3uL (0.20 – 0.90)
Eosinophils	0.56 x 10^3uL (0.00 – 0.90)
Basophils	0.10 x 10^3uL (0.00 – 0.20)
Platelets	333 x 10^3 /mmc (150 – 400)
Glycemia	78 mg/dL (74 -106)
Creatinine	0.31 mg/ dL (0.67 – 1.17)
Urea	20 mg /dL (10 – 50)

ALT	19 U/L (10 – 50)
AST	23 U/L (10 – 50)
LDH	570 U/L (208 – 378)
Folic Acid	14 ng/mL (2.0 -16.0)
Cobalamin	83.0 pg/mL (160.0 – 850.0)

Table S3. Concentration levels (µM) for calibrators and QC materials for LC-MS/MS analysis of hcy, mma and mca. SS1= 10mM, and SS2= 1mM.

Concentration (µM)	SS2 (µL)	SS1 (µL)	Whole Blood	Final Volume
0	0	0	1 mL	1 mL
2.5	2.5	0	997.5 µL	1 mL
5	5	0	995 µL	1 mL
10	10	0	990 µL	1 mL
50	0	5	995 µL	1 mL
100	0	10	990 µL	1 mL
QC LOW (5)	5	0	995 µL	1 mL
QC HIGH (25)	0	2.5	997.5 µL	1 mL

Table S4. Multiple Reaction Monitoring (MRM) functions and settings for detection of hcy, mma and mca are shown.

MRM Function	Time Window (min)	Analyte	Transitions (*m/z*)	Cone Volts	Collision Energy (eV)
1	0-9.00	hcy<br>$^2H_4$-hcy	192.20>90.20<br>196.20>94.20	40	15
2	0-9.00	mma<br>$^2H_3$-mma	231.20>119.20<br>234.20>122.40	20	9
3	0-9.00	mca 2But<br>$^2H_3$-mca 2But<br>mca 3But<br>$^2H_3$-mca 3But	319.20 > 143.20<br>322.20 > 146.20<br>375.20 > 199.20<br>378.20 > 202.00	20	18

Table S5. The Linearity criteria of the method were evaluated by analyzing the DBS samples fortified with increasing concentrations of hcy, mma and mca for five consecutive days. For each analytical session, the linear regression coefficient R2 was assessed. The method proved linear with $R^2 > 0.994$ for hcy, $R^2 > 0.995$ for mma, $R^2 > 0.998$ for mca. Average values, both for calibration parameters with the respective standard deviations (SD) and for R^2, are reported.

Analite	Concentration range (µM)	Calibration function (n=5) Y= a(±SD)x + b(±SD)	R^2 (mean)
**hcy**	2.5-100	Y= 92.57 (±10.1) x + 2.75 (±0.92)	0.996
**mma**	2.5-100	Y= 40.07 (±2.45) x + 0.76 (±0.69)	0.997
**mca**	2.5-100	Y= 49.00 (±1.47) x + 0.15 (±0.28)	0.998

Table S6. The method reproducibility, accuracy and precision were assessed, examining the Mean Value, Standard Deviation and CV% of low and high QC.

Analite	***Precision***			**Accuracy**		
**QC low**	**Mean value (n=4) (µM)**	**SD (n=4)**	**CV%**	**Nominal Value (µM)**	**Bias % (n=4)**	**SD (n=4)**
**hcy**	12.45	1.00	8.04	12.9	-3.48	0.08
**mma**	5.6	0.35	6.35	5.4	+3.70	0.06
**mca**	5.02	0.38	7.5	5.0	+0.5	0.075
**QC high**	**Mean value (n=4) (µM)**	**SD (n=4)**	**CV%**	**Nominal Value (µM)**	**Bias % (n=4)**	**SD (n=4)**
**hcy**	31.85	4.15	13.05	32.9	-3.19	0.13
**mma**	26.6	0.68	2.55	25.4	+4.72	0.03
**mca**	25.4	0.98	3.87	25.0	+1.6	0.04
